# Long term hemodialysis aggravates lipolytic activity reduction and very low density, low density lipoproteins composition in chronic renal failure patients

**DOI:** 10.1186/1471-2261-9-41

**Published:** 2009-08-26

**Authors:** Khedidja Mekki, Josiane Prost, Mustapha Remaoun, Jacques Belleville, Malika Bouchenak

**Affiliations:** 1 Laboratoire de Nutrition Clinique et Métabolique, Département de Biologie, Faculté des Sciences, Université d'Oran-31100 Es-Sénia, Algerie; 2 Upres Lipides & Nutrition, EA.2422. Faculté des Sciences Gabriel, Université de Bourgogne, 6 Bd Gabriel, 21000 Dijon, France; 3 Service de Néphrologie-Hémodialyse, Centre Hospitalo-Universitaire de Sidi Bel Abbès, Algerie

## Abstract

**Background:**

Dyslipidemia, particularly hypertriglyceridemia is common in uremia, and represents an independent risk factor for atherosclerosis.

**Methods:**

To investigate the effects of hemodialysis (HD) duration on very low density lipoprotein (VLDL) and low density lipoprotein (LDL) compositions and lipopolytic activities, 20 patients on 5 to 7 years hemodialysis were followed-up during 9 years. Blood samples were drawn at T0 (beginning of the study), T1 (3 years after initiating study), T2 (6 years after initiating study) and T3 (9 years after initiating study). T0 was taken as reference.

**Results:**

Triacylglycerols (TG) values were correlated with HD duration (r = 0.70, P < 0.05). An increase of total cholesterol was noted at T2 and T3. Lowered activity was observed for lipoprotein lipase (LPL) (-44%) at T3 and hepatic lipase (HL) (-29%) at T1, (-64%) at T2 and (-73%) at T3. Inverse relationships were found between HD duration and LPL activity (r = -0.63, P < 0.05), and HL activity (r = -0.71, P < 0.01). At T1, T2 and T3, high VLDL-amounts and VLDL-TG and decreased VLDL-phospholipids values were noted. Increased LDL-cholesteryl esters values were noted at T1 and T2 and in LDL-unesterified cholesterol at T2 and T3.

**Conclusion:**

Despite hemodialysis duration, VLDL-LDL metabolism alterations are aggravated submitting patients to a greater risk of atherosclerosis.

## Background

Post heparin plasma contains at least two lipolytic enzymes, lipoprotein lipase (EC 3.1.1.34, triacylglycerol-protein acyl hydrolase) and hepatic triacylglycerol lipase (EC 3.1.1.3, triacylglycerol acyl hydrolase). LPL is an extrahepatic enzyme and exerts its hydrolytic activity on circulating triacylglycerols (TG) carried by lipoproteins associated with the surface bound apolipoprotein (apo) C-II, a cofactor activator and involves the release of free fatty acids which are taken up by tissues [1].

Hepatic lipase is a hydrolytic enzyme, closely related, but not identical to LPL. It is synthesized by the liver parenchymal cells but acts at the luminal wall of the liver sinusoids, in analogy with LPL. HL catalyses the hydrolysis of triacylglycerols- (TG) and phospholipids- (PL) high density lipoprotein (HDL_2_) and TG-very low density lipoprotein (VLDL). There is also evidence that HL is mandatory for the final conversion of intermediary density lipoproteins (IDL) to low density lipoproteins (LDL) [1].

Atherosclerosis is recognized as the major cause of cardiovascular disease (CVD) in hemodialysis and it is responsible for 40% to 50% of deaths in this population [2]. Patients with cardiac surgery, on dialysis have a high risk of perioperative mortality and poor long term survival rates [3]. It has been suggested that dialysis itself could accelerate atherosclerosis and researchers agree that many patients enter dialysis with atherosclerosis, which can lead to a high risk of early mortality during the first years of dialysis [4].

Dyslipidemia, is often observed in patients with chronic renal failure (CRF), resulting in abnormal concentrations and composition of plasma lipoproteins. The prominent features of uremic dyslipidemia are an increase in plasma triglycerides in nearly all lipoproteins, and a reduction in high-density lipoprotein (HDL) cholesterol [5-7]. Hypertriglyceridemia is considered as an independent risk factor for CVD [8] and is explained in CRF by a defective catabolism of triglyceride rich lipoproteins by the lipolytic enzymes [9,10].

In hemodialysis patients, postheparin plasma LPL activity and HL activity have been reported to be reduced, while the apo CII/apo CIII ratio is decreased. A possible disturbance in both enzymes, accompanied by an increase in apo CIII in VLDL, results in a prolonged half-life of the VLDL particles, which may explain the observed hypertriglyceridemia in these patients [9-11].

However, the effects of long term hemodialysis on lipolytic activities are not be clarified. There are many controversies about the effects of dialysis duration on plasma lipid metabolism. Some authors [9,10,12] have found that, lipid and lipoprotein compositions do not appear to be influenced by dialysis duration. Authors have not establish any relationships between plasma TG levels and dialysis duration [9-13], whereas others have found correlation between hypertriglyceridemia, cholesterol, lecithin-cholesterol acyltransferase (LCAT) activity and hemodialysis duration [14-17].

Based on these observations, a 9 years longitudinal study was carried with the principal objective to evaluate the effects of hemodialysis (HD) duration on plasma lipids, lipoproteins and lipolytic activity alterations, in patients with chronic renal failure. This effect was assessed by correlation between duration of hemodialysis and lipolytic activities and lipoproteins composition.

## Methods

### Subjects

Twenty CRF patients were taken for maintenance hemodialysis during 9 years. Subjects were hemodialysed since 5 years at the beginning of the study. There were 6 women and 14 men (37 ± 12 years) with a plasma creatinine and urea values of 865 ± 231 μmol/L and 23 ± 4.85 mmol/L, respectively. CRF causes were unknown in 20% of patients and 80% had CRF secondary to chronic glomerulonephritis. All patients were treated with vitamins, phosphate, binding drugs and sodium bicarbonate for compensated anorexia. No patient was treated with drugs known to influence lipoprotein metabolism. Patients were dialysed 3 times weekly, using cuprophan membrane with a bicarbonate dialysate. The amount of delivered dialysis (Kt/V) was 1.20 ± 0.18 and patients presented a mean interdialytic body weight gain of 2.40 ± 1.17 kg, and received intravenously 0.5 mg.kg-1 BW of heparin at the beginning of the hemodialysis procedure and 0.25 mg.kg-1 BW each hour.

All patients were treated at the Nephrology-Hemodialysis ward of the University Hospital of Sidi Bel Abbes (west of Algeria). The purpose of this study was explained to the subjects and the investigation was carried out with their written consent. The experimental protocol was approved by the Committee for Research on Human Subjects of Sidi Bel Abbes Hospital.

### Assays

At the beginning of the study (T0) and at regular intervals during follow-up of the same patients, blood samples were drawn at four times, T0 (beginning of the study), T1 (3 years after initiating study), T2 (6 years after initiating study) and T3 (9 years after initiating study). Results of the T0 are taken as control. In all patients, blood was drawn from the dialysis fistule at each time after a 12-hour overnight fast. To obtain post-heparin plasma, blood samples were obtained 10 min after the intravenous injection of heparin (60 Units/kg). Plasma samples were collected by low speed centrifugation at 3000 × g at 5°C, for 15 min, preserved with 0.1% Na_2 _EDTA and 0.02% sodium azide and divided into aliquots stored at -70°C until analysis.

#### Lipid analysis and insulin concentration

triacylglycerols (TG) and total cholesterol (TC) were determined by specific colorimetric methods (Boehringer Mannheim Gmbh, Kits, Germany). ELISA (Enzyme Linked Immuno Sorbent) technique was used to determined insulin concentrations.

#### Lipolytic activity determination

plasma lipoprotein lipase (LPL) and hepatic lipase (HL) activities were measured with direct and selective methods [18] using as substrate a sonicated (3H)-trioleoglycerol emulsion stabilized by phosphatidylcholines. One mU of enzyme activity represented the release of 1 nmole fatty acid per min at 37°C. Enzyme activity was expressed as μmol of fatty acid. h^-1^. mL^-1 ^of plasma.

Hepatic lipase activity was expressed as μmol of released fatty acid. h^-1^. mL^-1 ^of plasma. The LPL activity was calculated as described by Nilsson-Ehle and Schotz [19]. One mU of enzymatic activity represented the release of 1 μmol fatty acid per min at 37°C.

#### Isolation and characterization of lipoprotein fractions

lipoproteins were isolated according to the method of Havel et al [20]. VLDL and LDL were isolated from total lipoproteins on a single spin discontinuous gradient [21], then were dialysed against 0.15 M NaCl + 0.04% Na_2 _EDTA, pH 7.4 at 4°C in Spectra/Por dialysis tubing (Spectrum Medical Industries, Los Angeles, CA), for 24 h.

In each lipoprotein fraction, protein contents were determined using bovine serum albumin (Sigma Chemical Company, St Louis, Mo) as standard [22]. Triacylglycerol, total cholesterol (TC) and unesterified cholesterol (UC) assays were performed by Boehringer reagent kits (Meylan, France). Esterified cholesterol values were obtained by difference between total cholesterol and unesterified cholesterol amounts. Total phospholipids (PL) were assayed by Wako reagent kit. VLDL was subjected to partial lyophilisation, followed by rapid delipidation with cold diethyl ether. For apolipoprotein (apo) C isolation, electrophoresis was performed [23]. Sodium dodecyl sulfate-polyacrylamide gel electrophoresis (SDS-PAGE), with 2.5–20% acrylamide, was carried out at 25 V for 18 h. After electrophoresis, apolipoproteins were stained with coomassie brillant blue G250. The proportions of each apolipoprotein were determined with the densitometer tracing at 600 nm (densitometer Model Profil 26, Sebia, Issy les Moulineaux, France). Results were expressed in arbitrary units (AU).

### Statistical analysis

Data are presented as the mean ± standard error, and were initially analysed by ANOVA test. Differences were assessed using Mann-Whitney test (non parametric). Levels of P < 0.05 were taken as significant. Linear regression analysis was used to determine correlation coefficients between LPL, HL activities, VLDL and LDL compositions and hemodialysis duration.

## Results

### Plasma triacylglycerol, total cholesterol and insulin concentrations

Patients showed a significant increase in triacylglycerol concentrations according to HD duration (Table 1). In hemodialysed patients, the TG values were higher 1.12-fold at T1 (P < 0.05), 1.31-fold at T2 (P < 0.05) and 1.63-fold at T3 (P < 0.01) than T0. No significant difference was noted in TC concentrations in patients at T1 compared to T0, but an increase by 14% and 33% was noted at T2 (P < 0.05) and T3 (P < 0.01), compared to T0. Insulin concentrations expressed in pg. L^-1 ^plasma were increased in hemodialysed patients, at T1 (P < 0.01), T2 (P < 0.001) and T3 (P < 0.01), compared to T0.

**Table 1:** Plasma triacylglycerol, total cholesterol, insulin, apo C-II and apo C-III concentrations and lipoprotein lipase and hepatic lipase activities, in hemodialysed patients.

	T0	T1	T2	T3
TG (mmol.L^-1^)	1.74 ± 0.17	1.96 ± 0.36*	2.29 ± 0.44*	2.84 ± 0.53**

TC (mmol.L^-1^)	3.56 ± 0.26	3.70 ± 0.22	4.07 ± 0.46*	4.76 ± 0.82**

Insulin (pg.L^-1^)	188 ± 23	209 ± 20**	223 ± 5***	200 ± 15**

LPL(μmol fatty acids.L^-1^. h^-1^)	8.68 ± 1.94	7.80 ± 1.91	7.60 ± 0.80	4.88 ± 1,20***

HL(μmol fatty acids.L^-1^. h^-1^)	4.02 ± 1.43	2,86 ± 1.40***	1,50 ± 0.40***	1,09 ± 2,85***

ApoCII (AU)	18.59 ± 5.66	17.51 ± 5.37	16.25 ± 4.45*	14.99 ± 4.12**

ApoCIII (AU)	24.43 ± 8.17	27.93 ± 7.92	36.65 ± 7.40***	50.51 ± 11.43***

Values are means ± SE. T0: Patients at the beginning of study, T1, T2 and T3: patients after 3, 6 and 9 years of the beginning of study, respectively. Comparison of the means was performed by ANOVA and using Mann-Whitney test: * T1, T2, T3 vs T0. * P < 0.05, ***P < 0.01, ***P < 0.001. LPL: Lipoprotein lipase, HL: Hepatic lipase, TG: Triacylglycerol, TC: Total cholesterol, Apo: Apolipoprotein, AU: Arbitrary units.

### Lipolytic activities

Lipolytic activities were decreased in patients according to HD duration (Table 1). A reduction in LPL activity by about 44% was noted at T3 (P < 0.001), compared to T0. HL activity was diminished by 29% at T1, 64% at T2 and 73% at T3 (P < 0.001), compared to T0.

### Plasma apo C-II and apo C-III concentrations

Reduced apo C-II and increased apo C-III concentrations (Table 1) were observed in patients, according to HD duration. Compared to T0, apo C-II values were decreased by 13% at T2 (P < 0.05) and 19% at T3 (P < 0.01). Apo C-III values were higher 1.5-fold at T2 (P < 0.001) and 2.06-fold at T3 (P < 0.001).

### VLDL and LDL amounts and compositions

**Figure 1:**
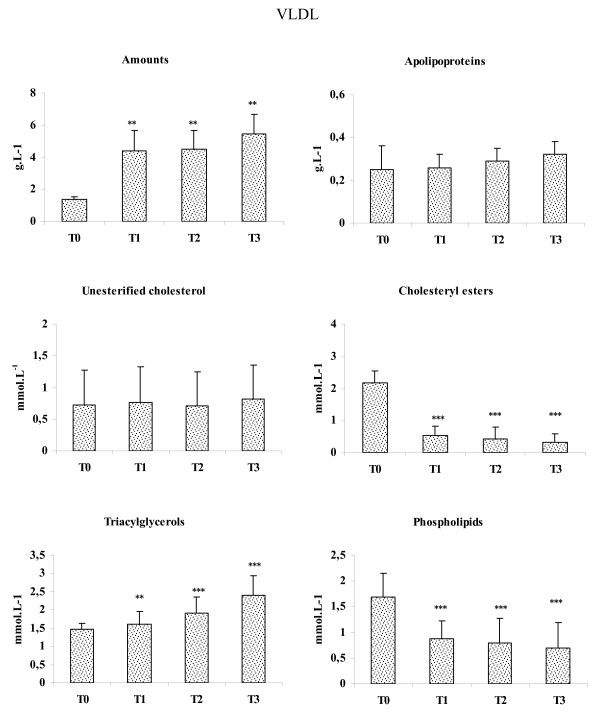
**Plasma VLDL amounts and compositions**. Values are means ± SE. T0: Patients at the beginning of study, T1, T2 and T3: patients after 3, 6 and 9 years of the beginning of study, respectively. Comparison of the means was performed by ANOVA and Mann-Whitney test: * T1, T2, T3 vs T0. * P < 0.05, ***P < 0.01, ***P < 0.001.

VLDL and LDL amounts, expressed in g.L^-1 ^plasma, were determined by adding their apolipoprotein and lipid components concentrations (apolipoproteins (apo) + triacylglycerols + unesterified cholesterol (UC) + cholesteryl esters (CE) + phospholipids (PL)). Plasma VLDL amounts were higher 3.2-fold at T1 (P < 0.01) and T2 (P < 0.01) and 3.9-fold at T3 (P < 0.01), compared to T0 (Fig 1). There was no significant difference in VLDL-apolipoproteins and VLDL-UC values according to HD duration, while, VLDL-CE amounts were diminished by 76%, 80% and 85% at T1 (P < 0.001), T2 (P < 0.001) and T3 (P < 0.001), respectively, compared to T0. VLDL-TG values increased according to HD duration and were more elevated at T1 (+9%) (P < 0.01), T2 (+30%) (P < 0.001) and T3 (+63%) (P < 0.001), compared to T0. VLDL-PL concentrations diminished according to HD duration, and values were progressively lowered at T1 (-49%) (P < 0.001), T2 (-53%) (P < 0.001) and T3 (-59%) (P < 0.001), compared to T0.

**Figure 2:**
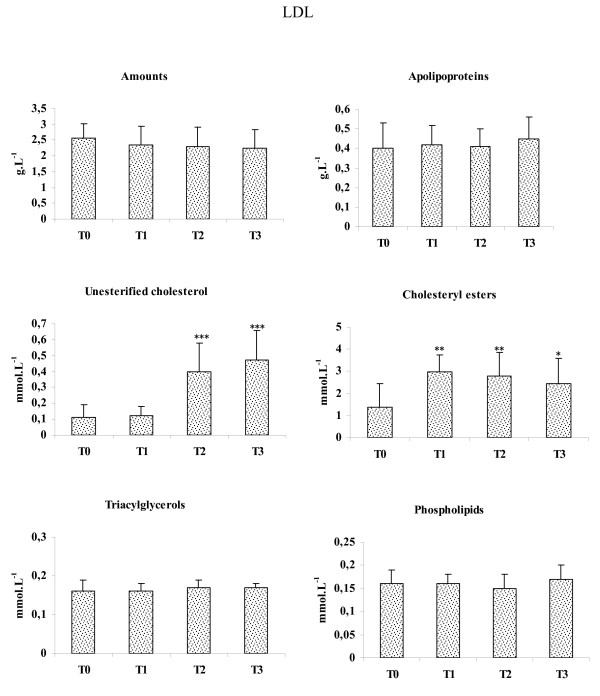
**Plasma LDL amounts and compositions**. Values are means ± SE. T0: Patients at the beginning of study, T1, T2 and T3: patients after 3, 6 and 9 years of the beginning of study, respectively. Comparison of the means was performed by ANOVA and Mann-Whitney test: * T1, T2, T3 vs T0. * P < 0.05, ***P < 0.01, ***P < 0.001.

There was no significant difference in plasma, LDL-amounts, -apolipoproteins, -TG and -phospholipids (Fig 2) values in patients according to HD duration. LDL-UC concentrations were 3.6- and 4.2-fold higher at T2 (P < 0.001) and T3 (P < 0.001), respectively than T0. A significant increase in LDL-CE was observed in patients and the values were 2.1-, 2- and 1.8-fold higher at T1 (P < 0.01), T2 (P < 0.01) and T3 (P < 0.05), respectively, compared to T0.

### Correlations analysis

Inverse relationships were found between HD duration and LPL activity (r = -0.63, P < 0.05), and HL activity (r = -0.71, P < 0.01). There was a positive correlation between HD duration and TG amounts (r = 0.64, P < 0.01), VLDL-amounts (r = 0.68, P < 0.01), VLDL-TG (r = 0.76, P < 0.01) and inverse relationship was noted between HD duration and VLDL-CE (r = -0.62, P < 0.01). No correlation was found between HD duration and LDL amounts and composition.

## Discussion

In order to determine the effects of long term hemodialysis treatment on plasma VLDL and LDL amounts and composition and lipolytic activities, a 9 years longitudinal study was carried in CRF patients, on regular and sufficient dialysis hemodialysed since 5 years at the beginning of the study (T0). In this study triacylglycerol concentrations were higher at each regular 3 years intervals and values were positively correlated with HD duration. Moreover, enhanced plasma TC values were found at T2 and T3. VLDL amounts and composition tended to be more increased with extensive hypertriglyceridemia and HD duration. Hypertriglyceridemia is an independent risk factor for CVD [8]. However, this hypertriglyceridemia seemed to be moderate compared with developed countries homologous values. This could be due to the Mediterranean diet consumed by our population, which was characterised by a high intake of vegetable proteins, complex carbohydrates, fibre and monounsaturated fatty acids [24].

Reduction of LPL activity in these patients was attributed to its increased essential inhibitor cofactor apo-CIII and diminished essential cofactor activator apo CII and hyperinsulinemia [17]. Insulin is a principal hormone stimulating LPL activity, a functional insulin deficiency could play an important role in this process in CRF [12]. Hyperinsulinemia disturb the activation of LPL and involve accumulation of VLDL and LDL [14].

In the present study, LPL and HL activities reduction was aggravated with HD duration. In contrast to some data [10,11,13], we found a negative correlation between HD duration, LPL and HL activities. LPL is activated by apo C-II and inhibited by apo C-III, which can also inhibit HL activity [10]. LPL activity was found [17] positively correlated with hyperinsulinemia, thus contributing, with increased apo C-III and reduced apo CII, to lowered LPL activity. Moreover, in this study, reduced apo C-II concentrations were noted at T2. On the other hand, the fact that our patients have adequate dialysis suggests that the observed disturbances are caused by other mechanisms. LPL hydrolyses TG carried by VLDL, and HL catalyses the hydrolysis of HDL_2_-TG and HDL_2_-PL [25]. HL activity reduction involves an increase in HDL_2_-TG and HDL_2_-PL. Indeed, high PL- and TG-HDL_2 _and TG-HDL_3 _were previously found in CRF patients [26]. Considering the role of LPL and HL in the lipolysis of plasma VLDL, impaired LPL and HL activities can account for hypertriglyceridemia [25], and increased VLDL concentrations [17]. These results are confirmed in this study since VLDL amounts and TG of VLDL and LDL were enhanced. Moreover, decreased VLDL-cholesteryl esters might be related to the low transfer of cholesteryl esters from HDL to VLDL and of TG in the opposite direction, probably related to a decreased cholesterol ester transfer protein (CETP) activity. VLDL-PL, which is the substrate for HL, was decreased and this was probably due to the low HL activity. Several mechanisms may explain the high plasma VLDL-amounts and VLDL-TG concentrations. The LPL synthesis was probably insufficient to hydrolyze the VLDL-TG, which would explain the high plasma VLDL-amounts and VLDL-TG concentrations in spite of unchanged LPL activity. Indeed, this low LPL synthesis can be attributed to the essential amino acids deficiency, knowing that our HD patients had a food intake characterized by reduced animal proteins (<10% of total protein intake).

On the other hand, hemodialysis requires repeated use of heparin, which reduces the reserve of the lipolytic enzymes, facilitating their release from the endothelium of blood vessels. This phenomenon could aggravate the LPL deficiency, these enzymes are not normally synthesized in uremic patients due to the resistance to the insulin action.

Literature data about the effect of HD duration on dyslipidemia generated by CRF are few and controversy. Our results differed from those of some authors who found that lipid and lipoprotein compositions did not appear to be influenced by dialysis duration in CRF patients [9-11]. However, these authors did not specify the dialysis duration in their patients, except by the statement that it was over 3 months. Ifudu et al [12] did not found any change in triglycerides and cholesterol concentrations according to increased HD duration, in patients hemodialyed for 10–24 years. However, the increase in triglyceride and cholesterol concentrations was positively correlated with the HD duration in the study of Sobh et al. [14]. Moreover, Paragh et al [15] showed that plasma triglycerides were positively correlated with HD duration, in patients hemodialysed during 8 to 181 weeks. In our study, plasma triglycerides, cholesterol, LPL, HL activities and VLDL-LDL compositions appeared clearly altered by HD duration. The evolutive lipid profile seemed to be more atherogenic and thus contributing to high cardiovascular risk.

## Conclusion

The results of this study demonstrates that in CRF patients treated by intermittent dialysis, long-term hemodialysis fails to correct dyslipidemia generated by CRF. Hypertriglyceridemia, increases of VLDL-amounts, VLDL-TG and VLDL-CE and reduction of LPL and HL activities are correlated with HD duration. Long term hemodialysis does not improve LDL-TG increase and LDL-PL decrease; moreover long term hemodialysis lowers apo C-II and enhances apo CIII and VLDL-CE. It can be suggested that patients on hemodialysis are particularly prone to atherosclerosis risk and that long term hemodialysis itself may worsen this condition. Dialysis can effectively reduce the accumulation of nitrogenous metabolites and correct disturbed fluid and electrolyte homeostasis, but patients on dialysis are still exposed to several of the metabolic consequences of renal failure. It could appear highly advantageous to reduce complication of long term dialysis patients with preventing modalities. Patients could benefit from decreased prevalence of cardiovascular and atherosclerotic diseases.

## Competing interests

The authors declare that they have no competing interests.

## Authors' contributions

MK: Study design, data collection, Biochemical and statistical analysis, literature search and manuscript preparation:

PJ: Biochemical analysis.

BM: Study design and manuscript preparation.

RM: Data collection.

BJ: Manuscript preparation.

## Pre-publication history

The pre-publication history for this paper can be accessed here:


